# Novel Receive Antenna Selection Scheme for Precoding-Aided Spatial Modulation with Lattice Reduction

**DOI:** 10.3390/s22093575

**Published:** 2022-05-07

**Authors:** Sangchoon Kim

**Affiliations:** Department of Electronics Engineering, Dong-A University, 37, Nakdong-Daero 550beon-gil, Saha-Gu, Busan 604-714, Korea; sckim@dau.ac.kr; Tel.: +82-51-200-7705

**Keywords:** antenna selection, multiple-input multiple-output (MIMO), precoding, zero-forcing (ZF), spatial modulation, lattice reduction

## Abstract

In this paper, a new receive antenna subset (RAS) selection scheme is proposed for precoding-aided spatial modulation (PSM). First, a lattice reduction (LR)-based precoder is employed instead of a conventional zero-forcing (ZF) precoder. It is analytically shown that a full diversity gain can be achieved by the LR-based ZF precoder without RAS selection. Then, an optimal LR-based RAS selection criterion is derived for the over-determined LR-based PSM systems, and a suboptimal selection algorithm is additionally presented. It is also shown that optimal and suboptimal RAS selection algorithms based on LR improve the BER performance of the LR-based PSM system. Further, the overall diversity order of the over-determined LR-based PSM systems with optimal LR-based RAS selection is analyzed. Finally, diversity analysis and simulation results show that the LR-ZF-based PSM system with optimal LR-based RAS selection outperforms the conventional ZF-based PSM system with conventional optimal RAS selection.

## 1. Introduction

A spatial modulation (SM) is a low-complexity multiple-input multiple output (MIMO) technique [1,2]. It not only utilizes the symbol constellation but also the indices of transmit antennas to carry information, while using a limited number of radio-frequency (RF) chains. Precoding-aided spatial modulation (PSM) has been designed as an emerging SM technique [3,4,5,6]. In a PSM scheme, the spatial position of each receive antenna is exploited as a source of information. It allows receiver side design with low-cost and low-complexity. The under-determined MIMO systems, where the number of receive antennas $N_{R}$ is equal to or smaller than that of transmit antennas $N_{T}$, are considered for ensuring precoding design.

Antenna selection techniques are considered to reduce the number of expensive RF chains and improve the system performance, while keeping spatial diversity gains [7,8]. Recently, receive antenna subset (RAS) selection [9,10] has been studied to enable the PSM in the over-determined MIMO systems $\left(N_{R}>N_{T}\right)$. In [9], the optimal exhaustive search and greedy incremental algorithms are presented to select an RAS for the PSM system. In [10], two classes of efficient RAS selection schemes have been proposed for the PSM system. However, a zero-forcing (ZF) precoder used in both [9,10] cannot offer a full diversity gain.

In [11], a lattice reduction (LR)-assisted precoding approach is considered for multiuser broadcast communications. It is shown in [12] that LR-aided MIMO broadcast decoding is able to capture a full receive diversity gain. The LR technique is able to generate a better conditioned channel matrix with more orthogonal and shorter basis vectors [13,14,15]. In [13,14,15,16,17], the LR is employed to enhance the performance of linear detectors such as ZF and minimum-mean-square-error (MMSE) equalizers in the conventional MIMO systems. To the best of the author’s knowledge, exploiting LR advantages in the PSM system has not been investigated to date.

In this paper, the concept of LR operation is first applied to the precoding scheme of the PSM system, and a new optimal RAS selection criterion based on the LR is presented. It is shown that the LR-assisted optimal RAS of selecting $N_{T}$ antennas among $N_{R}$ receive antennas in the LR-aided ZF-based PSM system with $N_{T}$ transmit antennas and $N_{R}$ receive antennas achieves a diversity order of $N_{T}(N_{R}-N_{T}+1)$. Meanwhile, it has been shown in [18] that the conventional optimal RAS of selecting $N_{T}$ antennas among $N_{R}$ receive antennas in the ZF-based PSM system with $N_{T}$ transmit antennas and $N_{R}$ receive antennas can obtain the diversity order of $\left(N_{R}-N_{T}+1\right)$. It is shown that the LR-aided ZF precoding can offer a better BER performance in comparison with the conventional PSM with RAS selection.

The remainder of this paper is organized as follows. In Section 2, an RAS selection scheme of the conventional PSM system is briefly presented. In Section 3, the PSM system with LR-based ZF precoding is introduced. In addition, the optimal LR-RAS selection and complexity-reduced LR-RAS selection algorithms are derived with their computational complexity analysis. Simulation results are presented in Section 4, where the diversity order analysis is also included. Finally, some conclusions are drawn in Section 5.

*Notations*: We use lower-case and upper-case boldface letters for vectors and matrices, respectively. Superscripts ${}^{*}$, ${}^{T}$, and ${}^{H}$ denote the complex conjugate, transposition, and Hermitian transposition, respectively. The notations Tr(⋅) and ${\left(\cdot \right)}^{-1}$ denote the trace and the inverse of a matrix, respectively. E[⋅], $\left|\cdot \right|$, and ${\left\|\cdot \right\|}_{F}$ represent the expectation, the absolute value, and the Frobenius norm, respectively. X(k,:) indicates the *k*-th row vector of a matrix X.

## 2. RAS Selection in Conventional PSM System

**Figure 1:**
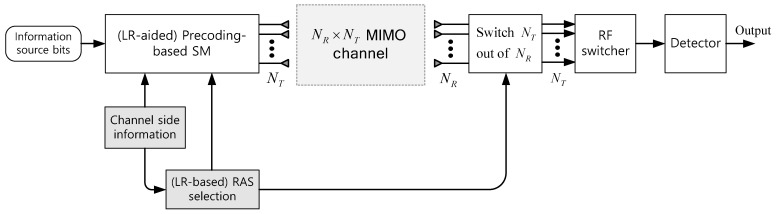
Block diagram of an (LR-aided) PSM MIMO TDD system with (LR-based) RAS selection.

Consider an over-determined MIMO time division duplex (TDD) system with $N_{T}$ transmit antennas and $N_{R}(N_{R}>N_{T})$ receive antennas as shown in Figure 1. The full channel matrix is given as $H\in C^{N_{R}\times N_{T}}$, which is the quasi-static channel matrix whose elements are independent and identically distributed (i.i.d.) circularly symmetric complex Gaussian random variables with zero mean and unit variance. We assume that the channel side information (CSI) of H is perfectly known at the transmitter and receiver. To design the PSM system, RAS selection is employed to obtain the selected channel. In this work, $N_{T}$ antennas out of $N_{R}$ receive antennas are assumed to be selected.

In TDD mode, CSI can be estimated by using the channel reciprocity property between uplink and downlink channels [19,20]. Pilot symbols may be transmitted from each receive antenna through an uplink channel in a round-robin manner among all the available receive antennas. The instantaneous CSI estimated at the transmitter can be utilized for LR-aided precoding and LR-based RAS selection. The indices of the selected RAS can be transmitted through a downlink channel or the RAS selection can be conducted by exploiting the CSI acquired at the receiver side.

The spatial modulated super-symbol vector is presented by $x\in C^{N_{T}\times 1}$, which can be expressed as $x=se_{r}$ where a symbol s with $E\left[{\left|s\right|}^{2}\right]=1$ is selected from the *M*-ary quadrature amplitude modulation (QAM) or phase-shift keying (PSK) constellation set and $e_{r}$ is the *r*-th column of the $N_{T}$-dimensional unit matrix. The super-symbol x is first precoded before transmission. Then, the transmit signal vector is given by βPx where $P\in C^{N_{T}\times N_{T}}$ is a precoding matrix and β is a power normalization factor used to ensure $E\left[{\left\|\beta Px\right\|}^{2}\right]=1$. In [3,6,9,10], the *ZF* precoding scheme has been applied to the PSM systems, and the precoding matrix is given as

(1) $$P_{ZF}=H_{S}^{H}{\left(H_{S}H_{S}^{H}\right)}^{-1}$$

where $H_{S}\in C^{N_{T}\times N_{T}}$ denotes the channel matrix obtained by an RAS selection algorithm.

The *k*-th received block signal at the receiver is described as

(2) $$y=\beta_{S}H_{S}Px+n=\beta_{S}x+n$$

where the power normalization factor related with the selected antenna subset is

(3) $$\beta_{S}=\sqrt{\frac{N_{T}}{Tr\left[{\left(H_{S}^{H}H_{S}\right)}^{-1}\right]}}$$

and $n\in C^{N_{T}\times 1}$ is an i.i.d. additive white Gaussian noise vector whose elements are the zero-mean circular complex white Gaussian noise component of a variance of $\sigma_{n}^{2}$. Then, the optimal RAS selection algorithm for the PSM system is expressed by [9]

(4) $$S^{*}=\underset{S\in \left\{S_{k},k=1,2,\cdots ,C\left(N_{R},N_{T}\right)\right\}}{\arg\;\max}\beta_{S}$$

where $S_{k}$ is the *k*-th enumeration of the set of all available $C\left(N_{R},N_{T}\right)$ antenna subsets. Here, $C\left(N_{R},N_{T}\right)$ is the total number of combinations of selecting $N_{T}$ antennas among $N_{R}$ receive antennas. In the receiver for the ZF-based PSM, the optimal maximum likelihood (ML) detector is given by

(5) $$\hat{x}=\underset{x}{\arg\;\min}{\left\|y-\beta_{S^{*}}x\right\|}^{2}$$

## 3. LR-Based RAS Selection in LR Aided PSM System

The ZF precoding-based SM system with $N_{T}\geq N_{R}$ (i.e., under-determined MIMO) whose transmit diversity order is well-known to be $N_{T}-N_{R}+1$ cannot provide the full diversity gain originating from multiple transmit antennas. This paper considers an LR-based PSM system (Figure 1) to achieve the maximum transmit diversity gain of $N_{T}$. LR is a process to find a new basis for the given lattice with basis vectors $\left\{h_{1},\;h_{2},\;\cdots ,\;h_{N_{T}}\right\}$, where $h_{i}$ is the *i*-th column vector of the full channel matrix H. The LR-reduced basis consists of nearly orthogonal and relatively short vectors. In this work, the LR is performed by a complex Lenstra–Lenstra–Lovász (CLLL) algorithm [14,15], which is the most common LR technique.

A specific channel matrix contained in the set of all possible $C\left(N_{R},N_{T}\right)$ antenna subsets is denoted by $H_{S}\in C^{N_{T}\times N_{T}}$. Then, the CLLL algorithm on the columns of $H_{S}^{H}$ is used to obtain the newly generated channel matrix

(6) $${\tilde{H}}_{S}=H_{S}^{H}T_{S}$$

where ${\tilde{H}}_{S}$ is a CLLL-reduced basis with approximately orthogonal columns, $T_{S}$ is an unimodular matrix, i.e., $\left|\det(T_{S})\right|=1$, and all elements of $T_{S}$ are Gaussian integers.

This work takes *LR*-aided *ZF* precoding at the transmitter for the PSM system into consideration. Then, the precoder based on *LR* is given by

(7) $$P_{LR-ZF}={\tilde{H}}_{S}^{}{\left({\tilde{H}}_{S}^{H}{\tilde{H}}_{S}\right)}^{-1}$$

The received signal vector in the LR-PSM system can be represented as

(8) $$\tilde{y}={\tilde{\beta }}_{S}{\left(T_{S}^{-1}\right)}^{H}x+n$$

where ${\tilde{\beta }}_{S}$ is a power normalization factor, which is expressed as

(9) $${\tilde{\beta }}_{S}=\sqrt{\frac{N_{T}}{Tr\left[{\left({\tilde{H}}_{S}^{H}{\tilde{H}}_{S}\right)}^{-1}\right]}}$$

Note that although the spatial modulated signal, x, in (8) consists of only a non-zero element, ${\left(T_{S}^{-1}\right)}^{H}x\in C^{N_{R}\times 1}$ given in the LR domain can contain two or more non-zero elements. Thus, the precoded signal may be received at more than one receive antenna in the LR domain.

The optimal ML detector for the LR-PSM can be obtained as

(10) $$\begin{array}{ll} \hat{x} & =\underset{x}{\arg\;\min}{\left\|\tilde{y}-{\tilde{\beta }}_{S}{\left(T_{S}^{-1}\right)}^{H}x\right\|}^{2}\\ & =\underset{x}{\arg\;\min}{\left\|\frac{\tilde{y}}{{\tilde{\beta }}_{S}}-\;{\left(T_{S}^{-1}\right)}^{H}x\right\|}^{2} \end{array}$$

The optimal RAS algorithm based on LR (called opt-LR-RAS) for the LR-PSM system can be straightforwardly formulated as

(11) $$\begin{array}{ll} S^{*} & =\underset{S\in \left\{S_{k},k=1,2,\cdots ,C\left(N_{R},N_{T}\right)\right\}}{\arg\;\max}{\tilde{\beta }}_{S}\\ & =\underset{S\in \left\{S_{k},k=1,2,\cdots ,C\left(N_{R},N_{T}\right)\right\}}{\arg\;\min}Tr\left[{\left({\tilde{H}}_{S}^{H}{\tilde{H}}_{S}\right)}^{-1}\right] \end{array}$$

IT is pointed out that the computational complexity of (11) to find an optimal LR-based RAS is very high due to an exhaustive search and CLLL operations. In this work, the LR is performed by a hardware-friendly CLLL algorithm named a fixed-complexity CLLL (fcCLLL) [21] to limit the worst-case complexity of CLLL. Furthermore, to perform LR-based RAS selection with reduced-complexity, a suboptimal LR-RAS selection algorithm is developed as a hybrid of norm and LR.

### 3.1. Proposed Suboptimal LR-RAS Selection Algorithm

A suboptimal LR-based RAS selection algorithm with less computational complexity than the optimal searching approach of (11) for the LR-PSM systems is presented, which is called subopt-LR-RAS and is summarized in Algorithm 1. It starts with an $N_{R}\times N_{T}$ full channel matrix H without LR. To avoid an exhaustive search, $\left(N_{T}-1\right)$ antennas among $N_{R}$ receive antennas are selected by computing a Frobenius norm of each row of the full channel matrix H, which is given by $C_{m}={\left\|{\underline{h}}_{m}\right\|}_{F}^{2}$, $m=1,2,\cdots ,N_{R}$, where ${\underline{h}}_{m}$ is the *m*-th row vector of the channel matrix H and then finding antenna indexes, $\left\{u(1),u(2),\cdots ,u(N_{T}-1)\right\}$, corresponding to $\left(N_{T}-1\right)$ largest values. After determining $\left(N_{T}-1\right)$ antennas, the resulting submatrix can be given by $H(u(1):u(N_{T}-1),:)$$\in C^{(N_{T}-1)\times N_{T}}$. To find the last antenna, the remaining antennas are added to the submatrix $H(u(1):u(N_{T}-1),:)$ one by one. That is, the $N_{T}\times N_{T}$ matrix can be formed as $H_{t-N_{T}+1}=\left[H(u(1):u(N_{T}-1),:);\;H(u(t),:)\right]\in C^{N_{T}\times N_{T}}$ where u(t), $t=N_{T},N_{T}+1,\cdots ,N_{R}$, indicates an antenna index of non-selected antennas. For each $H_{t-N_{T}+1}$, the fcCLLL operation is carried out to generate the new basis ${\tilde{H}}_{t-N_{T}+1}$.

Then, ${\tilde{\xi^{\prime }}}_{t-N_{T}+1}^{}=Tr\left({\left[{\tilde{H}}_{t-N_{T}+1}^{H}{\tilde{H}}_{t-N_{T}+1}\right]}_{}^{-1}\right)$, $t=N_{T},N_{T}+1,\cdots ,N_{R}$, can be computed. By assuming that the $Q_{k,t}$ matrix obtained from the fcCLLL is orthogonal, it results in ${\tilde{H}}_{t-N_{T}+1}^{H}{\tilde{H}}_{t-N_{T}+1}={\tilde{R}}_{t-N_{T}+1}^{H}{\tilde{R}}_{t-N_{T}+1}^{}$, which is used in line 6 of Algorithm 1. Here, ${\tilde{R}}_{t-N_{T}+1}^{}$ is the upper triangular matrix. The *fcCLLL* function in line 5 represents an fcCLLL algorithm without computing $\tilde{Q}$ and $\tilde{T}$ matrices in Algorithm 2, where ${\tilde{R}}_{t-N_{T}+1}(d,c^{\prime })$ is the $\left(d,c^{\prime }\right)$-th element of the upper triangular matrix ${\tilde{R}}_{t-N_{T}+1}^{}$. The last antenna can be found by $v=\underset{t^{\prime }=1,2,\cdots ,\left(N_{R}-N_{T}+1\right)}{\arg\;\min}{\tilde{\xi^{\prime }}}_{t^{\prime }}^{}$. Thus, the selected channel matrix can be expressed as $H_{S}=$ $\left[H(u(1):u(N_{T}-1),:);\;H(u(N_{T}-1+v),:)\right]\in C^{N_{T}\times N_{T}}$.
**Algorithm 1: Suboptimal LR-RAS Selection Algorithm**$$\begin{array}{cc} \; & \mathrm{Inputs}:\;H,\;\;\delta ,\;N_{R},\;\;N_{T},N_{iter}\\ \; & 1:\;\;C_{m}={∥\;{\underline{h}}_{m}∥}_{F}^{2},\;m=1,2,\cdots ,N_{R}\\ \; & 2:\;\;\left[\;V,\;\;u\;\right]=\mathrm{sort}\left\{\;C_{1},C_{2},\cdots ,C_{N_{R}}\right\}\;\mathrm{in}\;\mathrm{descending}\;\mathrm{order}\\ \; & 3:\;\;\mathrm{for}\;t=N_{T}:N_{R}\\ \; & 4:\;\;H_{t-N_{T}+1}=\left[\;H\left(u\left(1\right):u\left(N_{T}-1\right),:\right)\;\;\;;\;H\left(u\left(t\right),:\right)\;\right]\\ \; & 5:\;\;\left[\;{\tilde{R}}_{t-N_{T}+1}^{}\right]=fcCLLL\left(H_{t-N_{T}+1}^{H},\delta ,N_{iter}\right)\\ \; & 6:\;\;{\tilde{\xi^{\prime }}}_{t-N_{T}+1}^{}=Tr\left(\;{\left[\;{\tilde{R}}_{t-N_{T}+1}^{H}{\tilde{R}}_{t-N_{T}+1}\right]}_{}^{-1}\right)\\ \; & 7:\;\;\mathrm{end}\\ \; & 8:\;\;v=\underset{t^{\prime }=1,2,\cdots ,\left(N_{R}-N_{T}+1\right)}{\;\arg\;\min}\;{\tilde{\xi^{\prime }}}_{t^{\prime }}^{}\\ \; & 9:\;\;H_{S}=\left[\;H\left(u\left(1\right):u\left(N_{T}-1\right),:\right)\;\;\;;\;H\left(u\left(N_{T}-1+v\right),:\right)\;\right]\\ \; & \mathrm{Output}:\;H_{S} \end{array}$$

### 3.2. Computational Complexity

To evaluate the computational complexity, we take account of the number of real multiplications and the number of real summations [22,23,24,25]. Recall that the number of antennas selected from $N_{R}$ receive antennas is assumed to be $N_{T}$, which is equal to that of transmit antennas. From Algorithm 1, the complexity of the proposed suboptimal LR-based RAS selection algorithm in terms of real multiplications and summations, respectively, can be analyzed as

(12) $$N_{proposed}^{RM}=\left(N_{R}-N_{T}+1\right)\left(4N_{T}^{3}+8N_{T}^{2}+C_{fcCLLL}^{RM}\left(N_{T}^{}\right)\right)+N_{R}\left(2N_{T}\right)$$

(13) $$N_{proposed}^{RS}=\left(N_{R}-N_{T}+1\right)\left(4N_{T}^{3}+6N_{T}^{2}-2N_{T}^{}+C_{fcCLLL}^{RS}\left(N_{T}^{}\right)\right)+N_{R}\left(2N_{T}-1\right)$$

where the complexity of the fcCLLL algorithm in Algorithm 2 is given by
**Algorithm 2: fcCLLL without Computing** $\tilde{Q}$ **and** $\tilde{T}$ **Matrices**$$\begin{array}{cc} \; & \mathrm{Inputs}:\;H_{t-N_{T}+1}^{H},\delta ,N_{iter}\\ \; & (1)\;\;c=\mathrm{size}\;(\;H_{t-N_{T}+1}^{H},2)\;\\ \; & (2)\;\;\left[\;{\tilde{Q}}_{t-N_{T}+1},\;\;{\tilde{R}}_{t-N_{T}+1}\right]=QR\;\left(H_{t-N_{T}+1}^{H}\right)\;\\ \; & (3)\;\;\mathrm{for}\;n_{idx}=1:N_{iter}\;\\ \; & (4)\;\;\mathrm{for}\;c^{\prime }=2:c\;\\ \; & (5)\;\;\mathrm{for}\;d=c^{\prime }-1:-1:1\;\\ \; & (6)\;\;\mu =\;\lceil \;{\tilde{R}}_{t-N_{T}+1}\left(d,c^{\prime }\right)/{\tilde{R}}_{t-N_{T}+1}\left(d,d\right)\;\rfloor \;\;\\ \; & (7)\;\;\mathrm{if}\;\mu \neq 0\\ \; & (8)\;\;{\tilde{R}}_{t-N_{T}+1}\left(1:d,c^{\prime }\right)={\tilde{R}}_{t-N_{T}+1}\left(1:d,c^{\prime }\right)-\mu \;{\tilde{R}}_{t-N_{T}+1}\left(1:d,d\right)\;\\ \; & (9)\;\;\mathrm{end}\\ \; & (10)\;\;\mathrm{end}\\ \; & \left(11\right)\;\;\mathrm{if}\;\delta {\left|\;{\tilde{R}}_{t-N_{T}+1}\left(c^{\prime }-1,c^{\prime }-1\right)\;\right|}^{2}>{\left|\;{\tilde{R}}_{t-N_{T}+1}\left(c^{\prime },c^{\prime }\right)\;\right|}^{2}+{\left|\;{\tilde{R}}_{t-N_{T}+1}\left(c^{\prime }-1,c^{\prime }\right)\;\right|}^{2}\\ \; & \left(12\right)\;\;\mathrm{Swap}\;\mathrm{columns}\;c^{\prime }-1\;\mathrm{and}\;c^{\prime }\;\mathrm{in}\;{\tilde{R}}_{t-N_{T}+1}\\ \; & \left(13\right)\;\;\mathrm{norm}=\sqrt{\;{\left|\;{\tilde{R}}_{t-N_{T}+1}\left(c^{\prime }-1,c^{\prime }-1\right)\;\right|}^{2}+{\left|\;{\tilde{R}}_{t-N_{T}+1}\left(c^{\prime },c^{\prime }-1\right)\;\right|}^{2}}\;\\ \; & \left(14\right)\;\;\alpha ={\tilde{R}}_{t-N_{T}+1}\left(c^{\prime }-1,c^{\prime }-1\right)/\mathrm{norm}\;\;\\ \; & \left(15\right)\;\;\beta ={\tilde{R}}_{t-N_{T}+1}\left(c^{\prime },c^{\prime }-1\right)/\mathrm{norm}\;\;\\ \; & \left(16\right)\;\;\Theta_{t-N_{T}+1}^{}=\left[\;\alpha^{*}\;\beta^{*}\;;\;\;-\beta \;\;\;\alpha \;\right]\\ \; & \left(17\right)\;\;{\tilde{R}}_{t-N_{T}+1}\left(c^{\prime }-1:c^{\prime },c^{\prime }-1:c\right)=\Theta_{t-N_{T}+1}^{}\;{\tilde{R}}_{t-N_{T}+1}\left(c^{\prime }-1:c^{\prime },c^{\prime }-1:c\right)\;\\ \; & \left(18\right)\;\;\mathrm{end}\\ \; & \left(19\right)\;\;\mathrm{end}\\ \; & \left(20\right)\;\mathrm{end}\\ \; & \mathrm{Outputs}:\;{\tilde{R}}_{t-N_{T}+1} \end{array}$$

(14) $$C_{fcCLLL}^{RM}\left(N_{T}^{}\right)=N_{iter}\left(2N_{T}^{3}+N_{T}^{2}+49N_{T}^{}+60\right)+C_{QR}^{RM}\left(N_{T}^{}\right)$$

(15) $$C_{fcCLLL}^{RS}\left(N_{T}^{}\right)=N_{iter}\left(N_{T}^{3}+20N_{T}^{}-\frac{7}{3}\right)+C_{QR}^{RS}\left(N_{T}^{}\right)$$

where the *QR* decomposition is performed by the modified Gram-Schmidt *QR* factorization and thus its complexity is

(16) $$C_{QR}^{RM}\left(N_{T}^{}\right)=N_{T}^{3}+3N_{T}^{2}$$

(17) $$C_{QR}^{RS}\left(N_{T}^{}\right)=2N_{T}^{3}-\frac{1}{2}N_{T}^{2}-\frac{1}{2}N_{T}^{}$$

For the LR-based exhaustive RAS selection algorithm, the complexity is given by

(18) $$N_{exhaustive}^{RM}=C\left(N_{R},N_{T}\right)\left(4N_{T}^{3}+8N_{T}^{2}+C_{fcCLLL}^{RM}\left(N_{T}^{}\right)\right)$$

(19) $$N_{exhaustive}^{RS}=C\left(N_{R},N_{T}\right)\left(4N_{T}^{3}+6N_{T}^{2}-2N_{T}^{}+C_{fcCLLL}^{RS}\left(N_{T}^{}\right)\right)$$

It is noted that the matrix operations described in lines 5 and 6 of Algorithm 1 are repeated $\left(N_{R}-N_{T}+1\right)$ times instead of $C\left(N_{R},N_{T}\right)$, which significantly reduces the complexity of the proposed suboptimal LR-based RAS selection algorithm, especially for large $C\left(N_{R},N_{T}\right)$.

## 4. Simulation Results and Diversity Analysis

In this section, the optimal and suboptimal LR-based RAS selection algorithms proposed for the presented LR-PSM system with $N_{T}$ transmit antennas and $N_{R}(N_{R}>N_{T})$ receive antennas are evaluated through Monte Carlo simulations over Raleigh flat-fading channels. The signal-to-noise ratio (SNR) is defined by the symbol energy to the noise power spectral density ratio, i.e., $\eta =1/\sigma_{n}^{2}$. The QPSK modulation is assumed and the receiver is based on ML detection. Moreover, the fcCLLL algorithm for LR operation employs δ=1 and the iteration number of $N_{iter}=3$ for LR-RAS selection and $N_{iter}=5$ for signal detection. For the BER performance comparison, the following five PSM systems are considered.

(a) conventional ZF-based PSM without RAS selection (called ZF w/o RAS)
(b) conventional ZF-based PSM with optimal RAS selection (called opt-RAS-ZF) [9]
(c) LR-ZF-based PSM without RAS selection (called LR-ZF w/o RAS)
(d) LR-ZF-based PSM with optimal LR-aided RAS selection (called opt-LR-RAS-LR- ZF)
(e) LR-ZF-based PSM with suboptimal LR-aided RAS selection (called subopt-LR- RAS-LR-ZF)

In addition, in the plots, the BER reference curves are given as a form of $c/SNR^{G}$ with solid lines, where *c* is an appropriately selected positive constant and *G* denotes a diversity gain. Note that the diversity order can be employed to determine the slope of the BER curve in log-scale at high SNR ranges [26].

**Figure 2:**
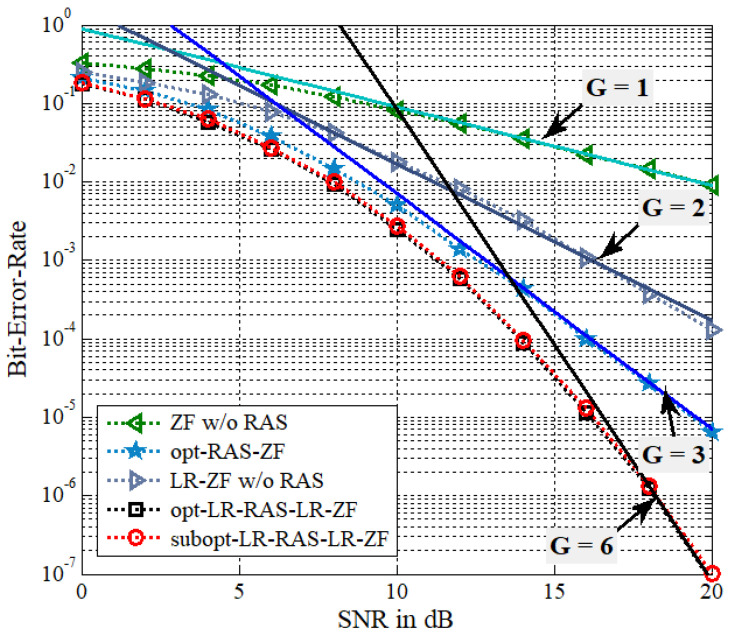
BER of the proposed algorithms for the LR-PSM system with $N_{T}=2$, $N_{R}=4$, and QPSK.

Figure 2 presents the simulated BER results of three antenna diversity systems of (b), (d), and (e) with $N_{T}=2$ and $N_{R}=4$. Thus, the spectral efficiency of 3 bps/Hz is assumed. Note that the number of selected receive antennas is equal to $N_{T}=2$. The other cases of (a) and (c), which have no antenna selection, correspond to the scenario of $N_{T}=2$ and $N_{R}=2$. Note that the PSM system with no RAS selection should meet the condition of $N_{R}\leq N_{T}$ to enable the precoding scheme. Thus, this work assumes that the number of receive antennas under the scenario without RAS selection is equal to the number of transmit antennas. It is found from BER curves of (a) and (c) that the LR-ZF-based PSM scheme outperforms the conventional ZF-based PSM. Using the similar method to [27,28], the transmit diversity order of ZF-based PSM without RAS selection can be easily obtained as $G=N_{T}-N_{R}+1$. It is clearly observed that ZF without RAS selection achieves the diversity order of G=1. To plot the BER reference curves, the constants selected for G = 1, 2, 3, and 6, are c = 0.9, 1.7, 7, and $8.3\times 10^{4}$, respectively. On the other hand, the LR-ZF-based PSM without RAS selection can capture the diversity order of $G=N_{T}$. Let us approximately analyze the transmit diversity order of the LR-ZF-based PSM scheme without RAS selection. The square of ${\tilde{\beta }}_{S}^{}$ can be re-expressed as

(20) $${\tilde{\beta }}_{S}^{2}=\frac{1}{E\left\{diag\left[{\left({\tilde{H}}_{}^{H}\tilde{H}\right)}^{-1}\right]\right\}}=E\left\{{\tilde{\beta }}_{r,S}^{2}\right\}$$

where

(21) $${\tilde{\beta }}_{r,S}^{2}=\frac{1}{{\left[{\left({\tilde{H}}_{}^{H}\tilde{H}\right)}^{-1}\right]}_{r,r}}={\tilde{h}}_{r}^{H}\tilde{F}{\tilde{h}}_{r}^{}$$

and ${\tilde{h}}_{r}^{}$ is the *r*-th column of $\tilde{H}$ and $\tilde{F}$ is an $N_{T}\times N_{T}$ non-negative Hermitian matrix formed from ${\tilde{h}}_{1}^{},\cdots ,{\tilde{h}}_{r-1}^{},{\tilde{h}}_{r+1}^{},\cdots ,{\tilde{h}}_{N_{T}}^{}$. Then, it is assumed for analysis purpose that the LR-reduced matrix $\tilde{h}$ consists of perfectly orthogonal column vectors. In this case, ${\tilde{h}}_{r}^{}$ is orthogonal to its projection on the subspace of $\tilde{F}$. Thus, the variable ${\tilde{\beta }}_{r,S}^{2}$ can be simplified as

(22) $${\tilde{\beta }}_{r,S}^{2}={\left\|{\tilde{h}}_{r}^{}\right\|}^{2}=\sum_{i=1}^{N_{T}}{\left|{\tilde{h}}_{r}^{\left(i\right)}\right|}^{2}$$

where ${\tilde{h}}_{r}^{\left(i\right)}$ is the *i*-th element of the vector ${\tilde{h}}_{r}^{}$. It means that the achievable transmit diversity order is evaluated as $N_{T}$. On the other hand, by taking the steps used in [18,29,30], the optimal RAS selection scheme of selecting $N_{T}$ antennas among $N_{R}$ receive antennas in the over-determined PSM system with $N_{T}$ transmit antennas and $N_{R}$ receive antennas can obtain the receive diversity gain of $N_{R}-N_{T}+1$. Then, by exploiting the analysis approach employed in [18], an achievable total diversity order of $N_{T}(N_{R}-N_{T}+1)$ can be obtained for the opt-LR-RAS-LR-ZF system. It is seen that the optimal LR-RAS selection algorithm of (11) can provide a significant improvement of BER performance compared to no antenna selection and thus the opt-LR-RAS-LR-ZF system outperforms the opt-RAS-ZF system. It is observed that the optimal exhaustive search RAS based on LR offers an extra receive diversity gain of 3, which is multiplied to the transmit diversity order $N_{T}=2$ of LR-ZF without RAS selection. Meanwhile, the optimal RAS selection without LR has only 2 more diversity gains than the ZF system without RAS selection. Furthermore, the BER results of the subopt-LR-RAS-LR-ZF are close to those of the opt-LR-RAS-LR-ZF system.

**Figure 3:**
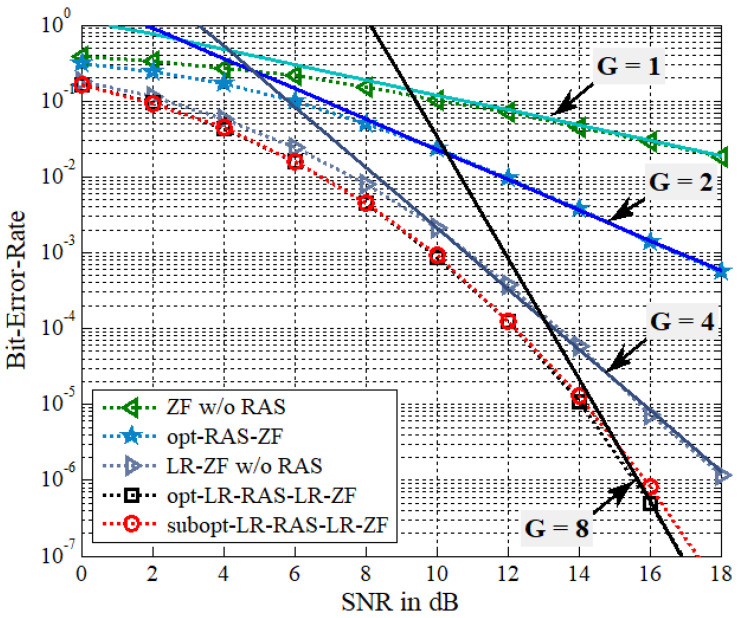
BER of the proposed algorithms for the LR-PSM system with $N_{T}=4$, $N_{R}=5$, and QPSK.

**Figure 4:**
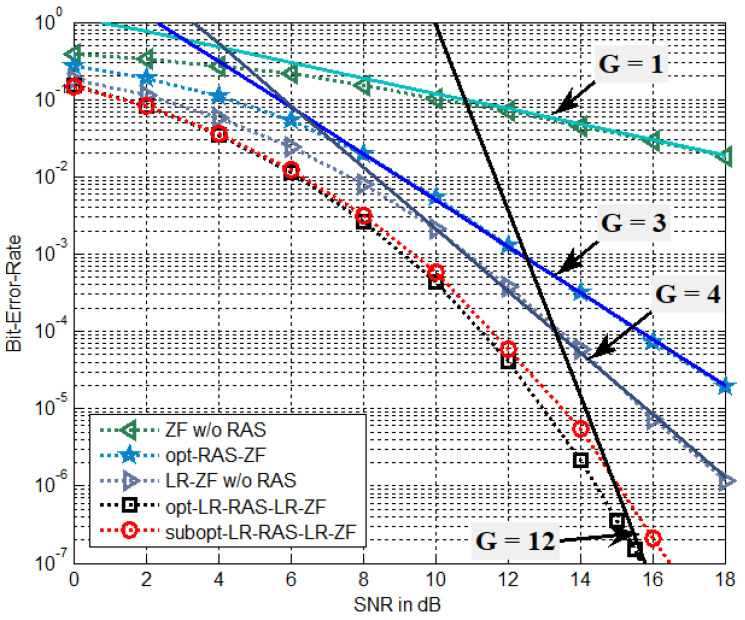
BER of the proposed algorithms for the LR-PSM system with $N_{T}=4$, $N_{R}=6$, and QPSK.

In Figure 3, the BER performance of antenna diversity systems of (b), (d), and (e) with $N_{T}=4$ and $N_{R}=5$ is compared to that of (a) and (c) with $N_{T}=4$ and $N_{R}=4$. Figure 4 employs the same simulation setup parameters as Figure 3 except for $N_{R}=6$. Thus, the spectral efficiency is given as 4 bps/Hz. In the BER reference curves of Figure 3, the constants used for the diversity gains, G = 1, 2, 4, and 8, are c = 1.2, 2.3, 21, and $3.35\times 10^{6}$, respectively. In Figure 4, the diversity orders of G = 1, 3, 4, and 12, use the constants of c = 1.2, 5, 21, and $9.2\times 10^{11}$, respectively. It is seen that the LR-ZF-PSM without RAS selection outperforms the ZF-PSM without RAS selection, which is due to different diversity gain. That is, the LR-ZF-PSM without RAS selection can achieve the full transmit diversity of $G=N_{T}=4$ while the diversity gain of the ZF-PSM is only $G=N_{T}-N_{R}+1=1$. Recall that no RAS selection implies that the scenario of $N_{T}=4$ and $N_{R}=4$. It is shown that the opt-LR-RAS-LR-ZF PSM system outperforms the LR-ZF-PSM without RAS selection and also has much better BER performance than the opt-RAS-ZF PSM system. Simulation results also confirm the analysis of diversity gains. It is also observed that the opt-LR-RAS-LR-ZF can achieve 4 and 8 more diversity gains, which are associated with $N_{R}=5$ and $N_{R}=6$, respectively, compared with the LR-ZF with no RAS selection, while the opt-RAS-ZF adds 1 and 2 more diversity gains, which correspond to $N_{R}=5$ and $N_{R}=6$, respectively, compared with the ZF with no RAS selection. Especially, note that the LR-ZF PSM system without RAS outperforms the opt-RAS-ZF PSM system for $N_{R}=6$ in Figure 4. On the other hand, the subopt-LR-RAS-LR-ZF exhibits slightly worse performance than the opt-LR-RAS-LR-ZF.

**Figure 5:**
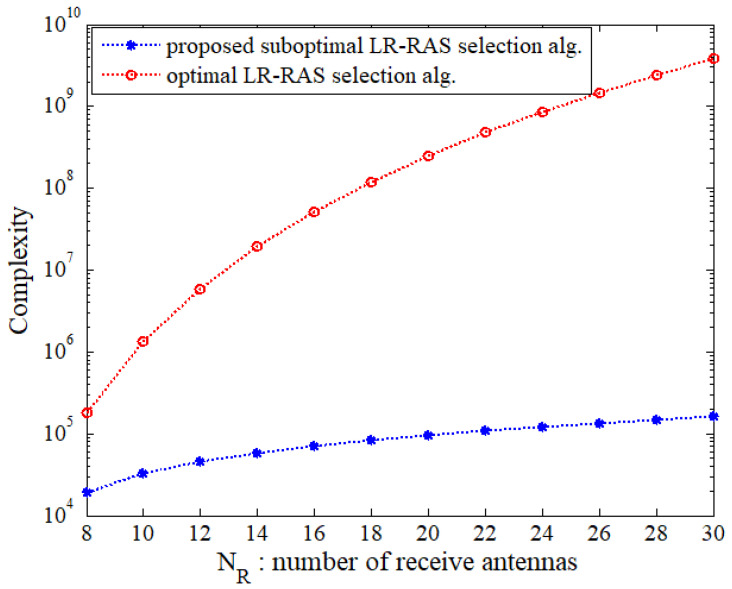
Complexity comparison of the proposed algorithm and optimal one for $N_{T}=6$ and $N_{iter}=3$.

**Figure 6:**
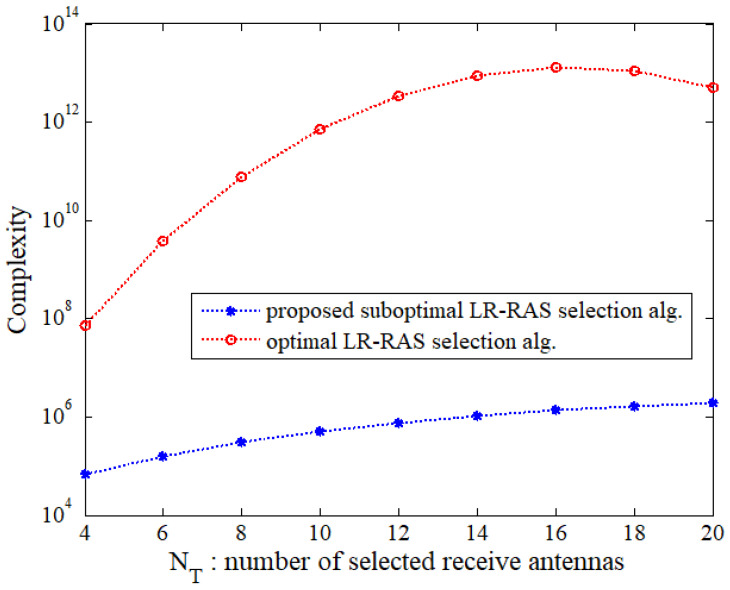
Complexity comparison of the proposed algorithm and optimal one for $N_{R}=30$ and $N_{iter}=3$.

Now, we compare the complexity of the proposed suboptimal LR-RAS selection algorithm with that of the optimal LR-RAS selection one. The complexity under the scenarios corresponding to Figure 2, Figure 3 and Figure 4 is presented in terms of RMs plus RSs in Table 1. It is observed that the proposed subopt-LR-RAS algorithm achieves much smaller complexity than the optimal one even for the given systems with relatively small number of $N_{T}$ and $N_{R}$. For $N_{T}=2$ and $N_{R}=4$, the complexity is reduced by about two times. As the number of antennas increases, the complexity reduction increases even further. In addition, the complexities of the algorithms used for the LR-ZF PSM system with large number of antennas are evaluated in Figure 5 in terms of the number of receive antennas for a fixed value of $N_{T}=6$ and in Figure 6 as a function of the number of selected receive antennas (equal to the number of transmit antennas) for a fixed value of $N_{R}=30$. In Figure 5 and Figure 6, a semi-log scale is used for *y*-axis and $N_{iter}=3$ is assumed. It is shown that as the antenna dimension increases, the rate of increase in the complexity of the proposed suboptimal algorithm is much slower than that of the optimal one. For the large antenna dimension, the complexity of the optimal one is huge, and thus the proposed suboptimal algorithm can tremendously reduce the complexity of the optimal one.

## 5. Conclusions

This paper considers the LR-based PSM system where an LR-aided ZF precoder is employed at the transmitter. It is shown that the LR-ZF-based PSM scheme without RAS selection can capture the maximum transmit diversity gain of $N_{T}$. Furthermore, we propose an optimal LR-based RAS selection algorithm for the over-determined LR-ZF-PSM system. Additionally, the optimal LR-RAS selection of choosing $N_{T}$ antennas among $N_{R}$ receive antennas in the over-determined LR-ZF-PSM system with $N_{T}$ transmit antennas and $N_{R}$ receive antennas can achieve the overall diversity order of $N_{T}(N_{R}-N_{T}+1)$, where $N_{T}$ is a transmit diversity gain and $\left(N_{R}-N_{T}+1\right)$ is a receive antenna selection diversity order. The LR-ZF-PSM scheme with optimal LR-RAS selection can achieve $N_{T}$ times larger diversity order than the conventional ZF-PSM system with optimal RAS selection. That is, the proposed LR-RAS selection algorithm yields the additional transmit antenna diversity gain compared to the conventional RAS selection and thus benefits the BER performance improvement in the LR-ZF-PSM system. If the number of antennas is large, the antenna diversity gain can also be large depending on the antenna dimension. To reduce the complexity of the optimal LR-RAS selection algorithm, a suboptimal LR-RAS selection algorithm combined with simple norm computations is presented. Even for a relatively small number of antennas, the complexity of the proposed algorithm is reduced by about more than two times compared to the optimal one. Especially under the large antenna dimension, the complexity reduction by the proposed suboptimal algorithm is huge.

## Figures and Tables

**Table 1:** Complexity of RMs plus RSs.

Parameters	Optimal	Suboptimal
$N_{T}=2,\;N_{R}=4,\;N_{iter}=3$	4920	2488
$N_{T}=4,\;N_{R}=5,\;N_{iter}=3$	12,915	5241
$N_{T}=4,\;N_{R}=6,\;N_{iter}=3$	38,745	7839

## Data Availability

Not applicable.
